# Risk Stratification and Adjuvant Chemotherapy for High‐Risk Stage IA Lung Adenocarcinoma: The Unmet Needs

**DOI:** 10.1111/1759-7714.15521

**Published:** 2024-12-21

**Authors:** Chen Shen, Haoran Liu, Bofei Li, Jiaming Wang, Yiyang Wang, Feichao Bao, Zhitao Gu, Wentao Fang

**Affiliations:** ^1^ Department of Thoracic Surgery, Shanghai Chest Hospital, School of Medicine Shanghai Jiao Tong University Shanghai China

**Keywords:** adenocarcinoma of lung, adjuvant chemotherapy, anaplastic lymphoma kinase, epidermal growth factor receptor, nomogram

## Abstract

**Introduction:**

To identify high‐risk patients for recurrence in resected stage IA lung adenocarcinoma and evaluate the impact of adjuvant chemotherapy (ACT) on their prognosis, as well as explore potential novel adjuvant therapies.

**Methods:**

Consecutive stage IA patients with ≥ 5% solid or micropapillary subtypes were analyzed. A nomogram was developed using Cox proportional hazards regression to predict recurrence‐free survival (RFS). In the high‐risk group after stratification, RFS was compared between patients receiving ACT and those under observation, as well as between patients with and without driver gene alterations.

**Results:**

This real‐world study included 1328 patients, with a 5‐year RFS of 79.0%. T stage and predominant subtype were independent risk factors for RFS. Patients with T1c or solid/micropapillary‐predominant tumors were stratified into a high‐risk group (*n* = 483) using the nomogram. A significant difference in 5‐year RFS was observed between the high‐ and low‐risk groups (73.6% vs. 84.3%, *p <* 0.001). Among high‐risk patients, sixty‐seven (13.8%) received ACT; however, there was no improvement in 5‐year RFS compared to observation alone (69.1% vs. 75.0%, *p =* 0.655). Testing rates for EGFR mutation and ALK fusion among high‐risk patients were only 52.4% and 43.9%, respectively, while mutation rates reached up to 55.7% and 9.4%, respectively. These molecular alterations exhibited numerically worse 5‐year RFS compared to wild‐type (EGFR mutation, 70.6% vs. 87.8%, *p* = 0.108; ALK fusion, 66.3% vs. 73.6%, *p* = 0.404), though not significant.

**Conclusions:**

ACT failed to meet the needs of stage IA patients with histological high‐risk features. Further exploration of effective adjuvant target therapies is warranted for this patient subgroup.

## Graphical abstract

We developed a predictive nomogram based on tumor T stage and histological predominant subtype in stage IA LUAD. And then we identified a patient group with increased risk of recurrence and found limited efficacy of ACT. Additionally, we observed insufficient testing rates but high mutation rates of driver genes, as well as their potential prognostic value.

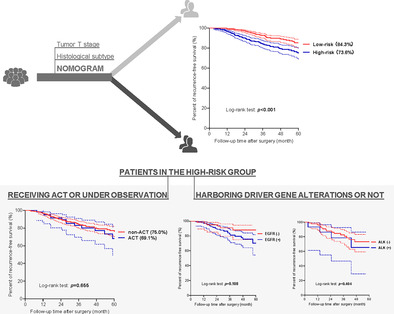

## 1 Introduction

Approximately 20%–30% of patients diagnosed with stage I non‐small cell lung cancer (NSCLC) face recurrence or mortality within 5 years following radical surgery [1, 2], which presents a significant unmet need for adjuvant treatment. Postoperative adjuvant chemotherapy (ACT) is recommended for patients with stage II‐IIIA NSCLC and stage IB high‐risk patients in the current guidelines [3], improving 5‐year survival rate by only 5.4% according to the lung adjuvant cisplatin evaluation (LACE) study [4]. For patients harboring driver gene alterations such as EGFR mutation and ALK fusion, target therapies have been proposed as the preferred choice of adjuvant treatment [3]. However, these recommendations do not extend to stage IA patients.

The risk factors for recurrence in patients with stage IA lung adenocarcinoma (LUAD) remain to be explored. Recent histological classification of lung cancer has highlighted the prognostic significance of histological subtypes. Specifically, solid and micropapillary subtypes, designated as high‐grade in the World Health Organization (WHO) 2015 classification [5], are associated with a significantly worse prognosis compared to other subtypes [6]. This adverse outcome persists even when high‐risk subtypes constitute as little as 5% of the tumor [7]. For stage IA LUAD patients harboring these histological high‐risk factors, the potential benefit of ACT remains an open question. Furthermore, the prognostic significance of molecular alterations including EGFR mutation and ALK fusion also continues to be debated [8, 9, 10]. The prevalence and prognostic relevance of molecular alterations in stage IA LUAD patients with histological high‐risk factors remain poorly defined.

The objective of this study is to identify a high‐risk patient group for recurrence in stage IA LUAD and investigate the potential benefit of ACT in these patients. Additionally, we seek to provide preliminary insights into the real‐world testing rates, mutation rates and prognostic value of EGFR mutation and ALK fusion. Ultimately, our study aims to contribute to optimizing postoperative treatment strategies in early‐stage LUAD.

## 2 Methods

### 2.1 Patient Selection and Study Design

This real‐world study was conducted at Shanghai Chest Hospital and was approved by the Institutional Review Board. We included all consecutive patients with completely resected T1N0M0 LUAD diagnosed between January 2013 and September 2020, presenting at least 5% solid or micropapillary subtypes. All participants underwent lobectomy and systematic lymph node dissection following the European Society of Thoracic Surgeons guidelines [11]. Pathological stage was determined based on the 8th edition of the tumor‐node‐metastasis classification by the American Joint Committee on Cancer staging manual [12]. Exclusion criteria included patients with invasive adenocarcinoma variants (including mucinous, colloid, fetal and enteric adenocarcinoma), multiple primary LUAD, or lost to follow‐up. Patients with high‐risk factors such as solid or micropapillary subtypes or lymphovascular invasion were indicated for ACT at the treating physician's discretion. The final decision regarding ACT administration was based on the physician's assessment of recurrence risk, patient performance status and patient preferences. Patients were then divided into two groups: those receiving three or four cycles of platinum‐based doublet chemotherapy (ACT group) and those not receiving ACT (observation group). The platinum drugs included cisplatin and carboplatin, and other drugs included pemetrexed, gemcitabine or vinorelbine. Patients who completed only one or two ACT cycles were also excluded. The study flowchart is shown in Figure S1.

### 2.2 Pathological Evaluation

Histological analyses were conducted according to the WHO 2015 classification by two experienced pathologists [5]. Histological subtypes were identified in 5% increments. The predominant subtype was determined by the highest percentage present. EGFR mutation and ALK fusion were identified via direct sequencing and either immunohistochemistry or fluorescence in situ hybridization.

### 2.3 Follow‐Up

Patients were followed at least every 6 months for the first 2 years after surgery and then once a year afterwards. Physical examination, serum tumor markers, chest CT scan, and ultrasonography of abdominal, cervical and supraclavicular regions were checked routinely. Brain magnetic resonance imaging, bone scans and positron emission tomography‐computed tomography were reserved when necessary.

### 2.4 Statistical Analysis

Continuous variables were reported as mean values (standard deviation), and categorical variables as number (percentage). Student's *t*‐test and Mann–Whitney test were used for continuous variables, while Chi‐square and Fisher's exact tests were used for categorical variables. Recurrence‐free survival (RFS) was defined as the time from surgery to the date of the earliest recurrence or death due to any cause or the last follow‐up. RFS was analyzed using the Kaplan–Meier method and log‐rank test. Multivariate Cox proportional hazards regression models were constructed to identify independent risk factors for RFS in the observation group. Hazard ratio (HR) and relative 95% confidence interval (CI) were calculated and depicted in forest plots. A nomogram was developed based on multivariate analysis to predict 5‐year RFS, with performance assessed by the concordance index (C‐index). Calibration of the nomogram was performed by comparing the predicted RFS with the actual observed RFS. Risk stratification, based on the nomogram, utilized an optimal cut‐off value identified via X‐tile software [13] to segregate all patients into high‐ and low‐risk groups. In the high‐risk group, RFS was compared between patients receiving ACT and those under observation, as well as between patients with driver gene alterations and those without. Statistical significance was set at *p* value less than 0.05. R software (version 4.3.1), IBM SPSS statistical software (version 25.0) and GraphPad Prism (version 8.0.2) were used for statistical analyses.

## 3 Results

### 3.1 Patient Demographics and Tumor Characteristics

A total of 1579 consecutive stage IA LUAD patients with at least 5% solid or micropapillary subtypes were enrolled. Among them, 164 patients received postoperative ACT. However, 24.4% of them (*n* = 40) completed only one or two cycles of ACT due to intolerance to chemotherapy and were thus excluded (Figure S1). Finally, 1328 patients remained for final analysis, and their clinicopathological characteristics were summarized in Table 1. ACT was conducted in 124 patients (9.3%). Compared with the observation group, the ACT group contained significantly more T1c tumors, more solid or micropapillary predominant subtypes and more lymphovascular invasion (all *p* < 0.05) (Table 1).

**TABLE 1:** Clinicopathological characteristics of stage IA lung adenocarcinoma patients with at least 5% solid or micropapillary subtypes.

Variables	Total (*n* = 1328)	Observation group (*n* = 1204)	ACT group (*n* = 124)	*p*
Sex				0.083
Female	682 (51.4)	628 (52.2)	54 (43.5)	
Male	646 (48.6)	576 (47.8)	70 (56.5)	
Age, mean (SD)	59.0 (9.2)	59.2 (9.3)	58.2 (9.2)	0.259
Smoking history				0.342
Never	905 (68.1)	827 (68.7)	80 (64.5)	
Ever	423 (31.9)	377 (31.3)	44 (35.5)	
T stage				< 0.001
T1a/T1b	1016 (76.5)	937 (77.8)	79 (63.7)	
T1c	312 (23.5)	267 (22.2)	45 (36.3)	
Predominant subtype				0.005
Lep/Pap/Aci	1108 (83.4)	1016 (84.4)	92 (74.2)	
Sol/Mip	220 (16.6)	188 (15.6)	32 (25.8)	
LVI				0.005
No	1285 (96.8)	1171 (97.3)	114 (91.9)	
Yes	43 (3.2)	33 (2.7)	10 (8.1)	
EGFR mutation				0.475^a^
Wild	282 (21.2)	247 (38.6^a^)	35 (42.7^a^)	
Mutated	440 (33.1)	393 (61.4^a^)	47 (57.3^a^)	
Unknown	606 (45.6)	564	42	
ALK fusion				0.296^a^
Wild	522 (39.3)	479 (91.8^a^)	43 (87.8^a^)	
Fusion	49 (3.7)	43 (8.2^a^)	6 (12.2^a^)	
Unknown	757 (57.0)	682	75	

Abbreviations: ACT, adjuvant chemotherapy; Lep/Pap/Aci, lepidic, papillary or acinar predominant; LVI, lymphovascular invasion; Sol/Mip, solid or micropapillary predominant.

^a^ Calculated among patients receiving testing.

### 3.2 Survival Analysis

**FIGURE 1:**
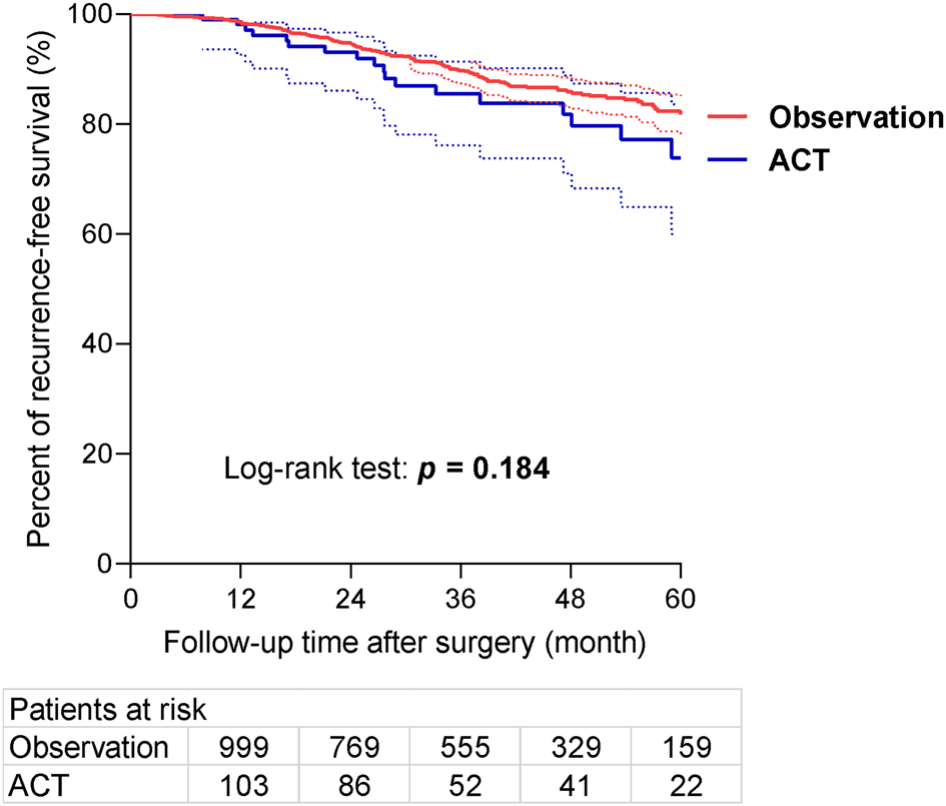
Kaplan–Meier curves for patients receiving ACT or under observation (*n* = 1328). ACT did not improve survival for the overall patient population (5‐year recurrence‐free survival, ACT vs. observation, 75.5% vs. 80.3%, *p* = 0.184). ACT, adjuvant chemotherapy.

**FIGURE 2:**
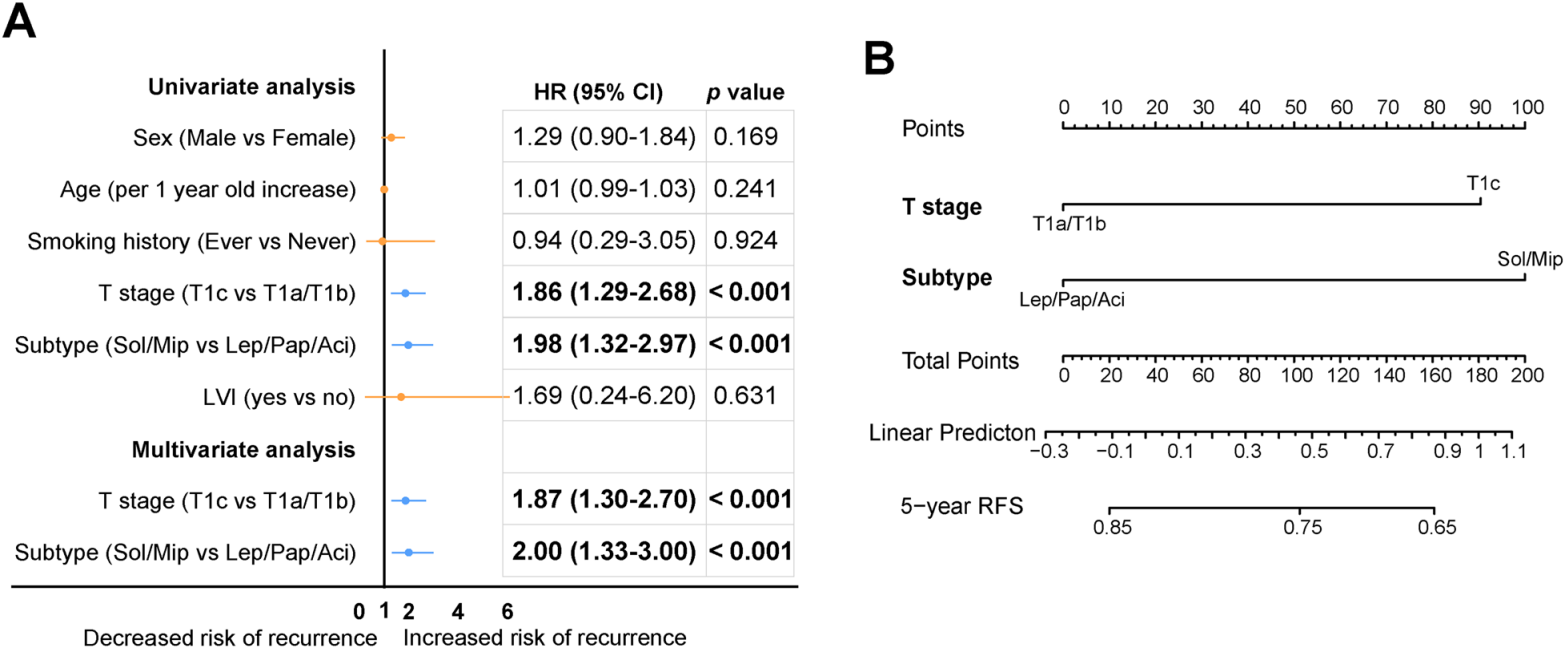
(A) Forest plots of risk factors for RFS in univariate and multivariate cox regression analysis. (B) The established nomogram for predicting 5‐year RFS. Sol/Mip, solid or micropapillary predominant; Lep/Pap/Aci, lepidic, papillary or acinar predominant; HR, hazard ratio; CI, confidence interval; RFS, recurrence‐free survival.

Median follow‐up was 55.6 months, ranging from 3.0 to 114.2 months. Overall 5‐year RFS was 79.0% (95% CI: 75.5%–82.5%). Five‐year RFS was similar between the ACT and observation groups (75.5% vs. 80.3%, *p =* 0.184) (Figure 1). In the observation group, tumor T stage (HR = 1.87, 95% CI: 1.30–2.70, *p* < 0.001) and histological predominant subtype (HR = 2.00, 95% CI: 1.33–3.00, *p* < 0.001) were identified as independent risk factors for RFS in multivariable analysis (Figure 2A).

### 3.3 Risk Stratification

**FIGURE 3:**
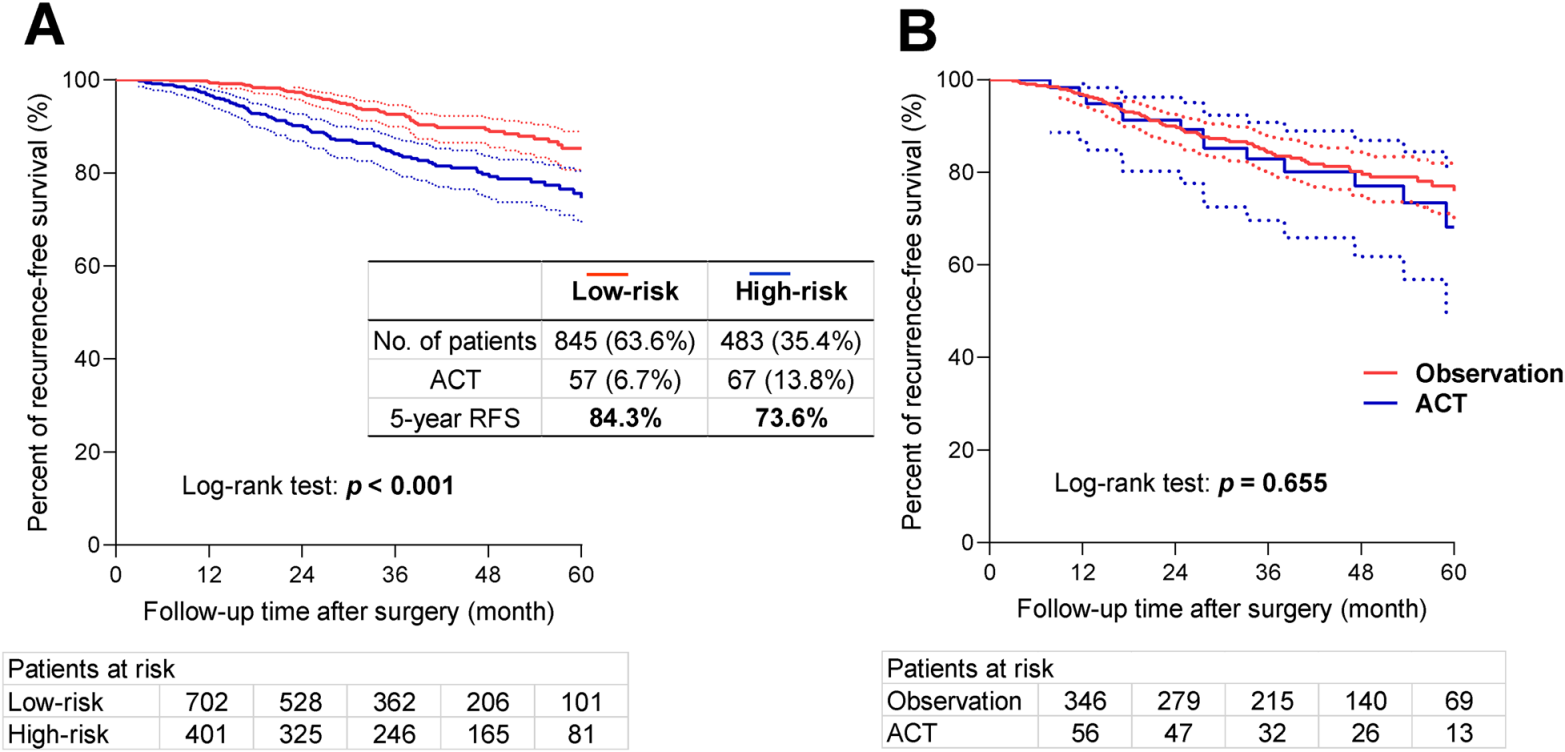
(A) Kaplan–Meier curves were well stratified by risk groups (5‐year RFS, high‐risk group vs. low‐risk group, 73.6% vs. 84.3%, *p <* 0.001). (B) Kaplan–Meier curves for high‐risk patients receiving ACT or under observation (*n* = 483). ACT did not improve survival for these high‐risk patients (5‐year RFS, ACT vs. observation, 69.1% vs. 75.0%, *p =* 0.655). RFS, recurrence‐free survival; ACT, adjuvant chemotherapy.

A nomogram including tumor T stage and histological predominant subtype was presented in Figure 2B. The nomogram exhibited good predictive performance, with a C‐index of 0.685 (95% CI: 0.659–0.711). The calibration curves showed good agreement between prediction and observation in the probability of 5‐year RFS (Figure S2). Risk score based on the nomogram was employed in all patients. Patients with T1c tumors or with solid or micropapillary predominant subtypes were divided into a high‐risk group, and those without were into a low‐risk group, with cut‐off score (score ≥ 90) generated using X‐tile software. The high‐risk group (*n* = 483), which accounted for 35.4% of the overall patient population, had significantly lower 5‐year RFS compared with the low‐risk group (73.6% vs. 84.3%, *p <* 0.001) (Figure 3A).

### 3.4 The Influence of ACT on RFS

In the high‐risk group (*n* = 483), 67 patients (13.8%) received postoperative ACT, which showed similar clinicopathological characteristics to those under observation (Table S1). Instead of improving survival for these high‐risk patients, ACT even exhibited a tendency towards worse prognosis compared to observation alone (5‐year RFS, ACT vs. observation, 69.1% vs. 75.0%, *p =* 0.655) (Figure 3B).

### 3.5 Driver Gene Alterations and Survival

**FIGURE 4:**
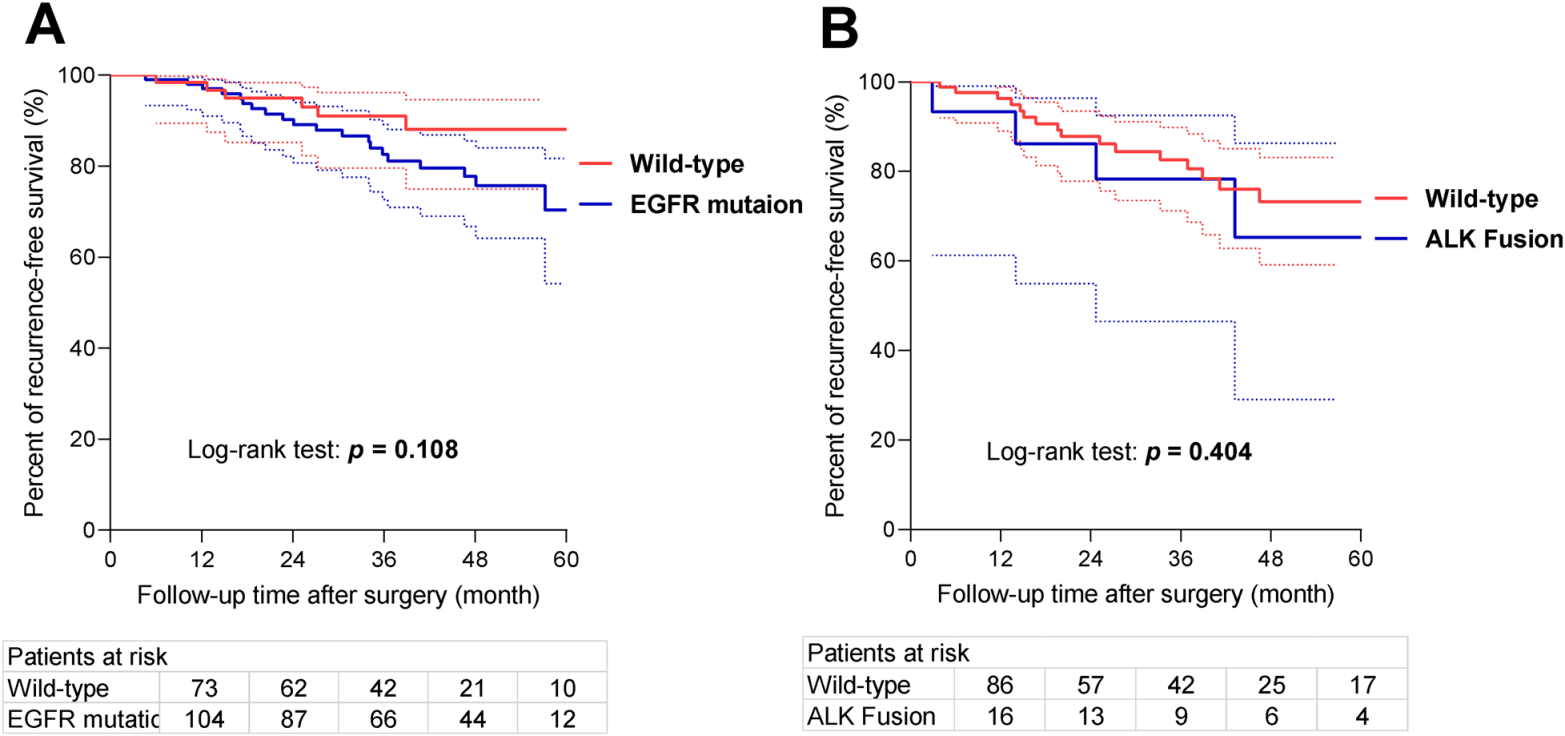
Kaplan–Meier curves for patients in the high‐risk group with different status of EGFR mutation or ALK fusion. (A) Five‐year recurrence‐free survival (RFS) tended to be lower in patients with EGFR mutation than those with wild‐type (70.6% vs. 87.8%, *p =* 0.108). (B) Five‐year RFS tended to be lower in patients with ALK fusion than those with wild‐type (66.3% vs. 73.6%, *p =* 0.404).

EGFR mutation and ALK fusion status were assessed in only 52.4% (*n* = 253) and 43.9% (*n* = 212) of patients in the high‐risk group, respectively. But among them, 141 patients (55.7%) harbored EGFR mutation, with 95.0% (*n* = 134) exhibiting either EGFR exon 19 deletions or L858R mutation in exon 21. The presence of ALK fusion was detected in 9.4% of patients (20 out of 212). Patients with EGFR mutation showed a numerically worse survival, although the difference was not statistically significant (5‐year RFS, EGFR mutation vs. wild‐type, 70.6% vs. 87.8%, *p =* 0.108) (Figure 4A). Similarly, patients with ALK fusion also showed a trend towards reduced survival (5‐year RFS, ALK fusion vs. wild‐type, 66.3% vs. 73.6%, *p =* 0.404) (Figure 4B).

## 4 Discussion

This real‐world study of stage IA patients with at least 5% high‐grade subtypes revealed the complex landscape of managing postoperative adjuvant therapy in early‐stage LUAD. We developed a predictive nomogram based on tumor T stage and histological predominant subtype, which could assist in identifying high‐risk patients who require adjuvant treatment. However, ACT failed to demonstrate any improvement in 5‐year RFS for either the overall or the high‐risk patient populations, and even showed numerically worse prognosis compared to observation alone, presenting the unmet needs in clinical practice. Furthermore, our investigation into driver gene alterations, particularly EGFR mutation and ALK fusion, indicated the significance of molecular testing and suggested the potential for target therapies to improve prognosis.

Recurrence remains a concern even after complete resection in patients with stage IA LUAD; therefore, it is imperative to identify high‐risk patient subgroups for recurrence. In this study, we developed a nomogram and stratified a group of high‐risk patients based on tumor T stage and histological predominant subtype, revealing significantly lower 5‐year RFS compared to the low‐risk group (73.6% vs. 84.3%, *p* < 0.001), indicating an important need for adjuvant treatment. Lymphovascular invasion was not included in the model (HR = 1.69, *p* = 0.631) primarily due to limited sample size. This straightforward yet practical nomogram showed good discrimination performance (C‐index of 0.685). However, the C‐index is relatively lower compared to some previous studies [14, 15], as this model was based on selected patients who had at least 5% high‐risk subtypes and did not include preoperative radiological information.

It is widely acknowledged that postoperative ACT may not confer any survival advantage across the entire population of patients with stage IA NSCLC and, to some extent, may have detrimental effects. The LACE study reported that 24% of treated patients were unable to complete at least 3 cycles of ACT, mainly because of toxicity or patient refusal [4]. In the EVAN study [16], grade 3 or worse adverse events occurred in 26% of patients in the ACT group versus 12% in the erlotinib group. Similarly, this real‐world study observed a substantial proportion (24.4%) of patients who could not complete the planned number of cycles of ACT due to intolerance of toxicity. As for stage IA patients with histological high‐risk factors, it is still under debate whether ACT can improve their prognosis [17, 18, 19, 20, 21]. We performed a literature search on the influence of ACT on stage IA NSCLC patients with histological high‐risk factors (Table S2) [17, 18, 19, 20, 21]. Some articles provided limited evidence supporting ACT utilization in specific subgroups of stage IA patients, including those with micropapillary‐predominant or poorly differentiated tumors [18, 19]. However, these studies suffered from either too small sample size or inadequate statistical methods to control bias. In another two large‐scale American national database analysis [20, 21], Luan et al. and Pathak et al. used various statistics methods and found that ACT did not improve survival for stage IA patients with poorly differentiated histological findings, which is in accordance with our study. In this largest single‐center real‐world cohort to date, we observed that ACT did not markedly influence 5‐year RFS, either in the overall population or among high‐risk patients after risk stratification, compared to those under observation. There still remain the unmet needs for adjuvant treatment in stage IA patients with histological high‐risk factors.

Beyond ACT, the emergence of adjuvant target therapies presents promising avenues for early‐stage LUAD treatment. Based on the ADAURA trial [22], osimertinib is now recommended as an adjuvant therapy for resected EGFR‐mutated stage IB‐IIIA NSCLC [3]. In the recently released ALINA study [23], adjuvant alectinib significantly improved survivals in patients with resected stage II‐IIIA NSCLC harboring ALK fusion. Several retrospective studies have reported the effectiveness of adjuvant EGFR tyrosine kinase inhibitor therapy in reducing recurrence rates among high‐risk stage IA patients [24, 25]. For example, a recent study showed that adjuvant aumolertinib therapy might improve 2‐year disease‐free survival in stage IA patients with at least 5% high‐grade subtypes [24], without any occurrence of grade 3 or worse adverse events, indicating the potential of target therapies in addressing the needs for effective and precise adjuvant treatment for high‐risk stage IA patients.

In this real‐world study, a relatively high percentage (55.7%) of patients in the high‐risk group were found to harbor EGFR mutation. Additionally, the prevalence of ALK fusion was 9.4%, which is higher than the overall frequency (5.1%) reported in a prospective study of Chinese lung cancer patients [26]. This difference can be attributed to the significantly higher prevalence of ALK fusion in high‐grade subtypes [27, 28]. The prognostic significance of EGFR mutation and ALK fusion in early‐stage LUAD patients remains uncertain, and recent studies have focused on specific patient groups. For instance, Ito et al. found that EGFR mutation was related to poor prognosis among stage I patients with solid or micropapillary predominant subtypes [8]. Similarly, some studies have suggested that ALK‐positive status is associated with worse prognosis compared with wild‐type [29, 30]. In our study focusing on high‐risk patients with solid/micropapillary predominant or T1c tumors, carriers of EGFR mutation (70.6% vs. 87.8%, *p =* 0.108) and ALK fusion (66.3% vs. 73.6%, *p* = 0.404) tended to exhibit a worse 5‐year RFS compared to those with wild‐type; however, statistical significance was not achieved mainly due to small sample size and low testing rates for EGFR and ALK status in these stage IA patients (52.4% and 43.9% respectively). The observed low testing rates during the study period were primarily attributed to a limited understanding of the prognostic significance of driver genes and inadequate awareness regarding the needs for adjuvant precision treatment in stage IA patients with histological high‐risk subtypes. In contrast, the real‐world testing rates for these molecular alterations could reach up to 70%–80% in advanced NSCLC [31, 32]. Moreover, it has been reported that availability of molecular testing before first‐line therapy is associated with significantly better survival among patients with advanced nonsquamous NSCLC [32], indicating the importance of molecular testing in advanced‐stage disease. In this study, we found that completion of molecular testing might also be important in postoperative early‐stage LUAD, even among stage IA patients since driver gene alterations were common and potentially indicative of worse prognosis. Considering these findings and the reported high efficacy and low toxicity profile with target therapies [22, 23, 24], insufficient molecular testing might hinder accurate prediction of recurrence risk and guidance for optimal postoperative treatment decisions in high‐risk stage IA patients.

There were certain limitations in this study. First, it was retrospective in nature with unavoidable internal biases. Second, other driver genes and molecular markers, such as KRAS, BRAF and programmed cell death ligand 1, were not included because they were not routinely tested in stage IA patients during the study period. However, our study provided valuable information on the testing rates, prevalence and potential prognostic significance of common driver gene alterations among high‐risk stage IA patients. Third, despite being the largest single‐center real‐world patient cohort reported to date, the case volume was still relatively small and represented experiences from a single center. Conducting multi‐center retrospective studies with longer follow‐up periods and prospective clinical trials on adjuvant therapy in high‐risk stage IA LUAD would be worthwhile for further validation of our findings.

In conclusion, we identified a patient group in stage IA LUAD with increased risk of recurrence after surgery and found limited efficacy of ACT, presenting the unmet needs within this population. Additionally, we observed insufficient testing rates but high mutation rates of driver genes, as well as their potential prognostic value. These findings emphasize the significance of molecular testing and provide valuable insights for postoperative management strategies in high‐risk stage IA LUAD.

## Author Contributions

**Chen Shen:** conceptualization, methodology, software, validation, formal analysis, investigation and writing – original draft. **Haoran Liu:** investigation and data curation. **Bofei Li:** methodology, software and validation. **Jiaming Wang:** investigation. **Yiyang Wang:** conceptualization, methodology and resources. **Feichao Bao:** validation and visualization. **Zhitao Gu:** resources and supervision. **Wentao Fang:** conceptualization, methodology, writing – review and editing, supervision, project administration and funding acquisition.

## Conflicts of Interest

The authors declare no conflicts of interest.

## Supporting information


Data S1.

**Figure S1.** Study cohort flowchart. LUAD, lung adenocarcinoma; ACT, adjuvant chemotherapy.

**Figure S2.** The calibration curves for predicting 5‐year RFS. Nomogram‐predicted RFS is plotted on the *x* axis; actual RFS is plotted on the *y* axis. A closer alignment of the drawn line with the diagonal indicates a better calibration model. RFS, recurrence‐free survival.

## Data Availability

The data that support the findings of this study are available on request from the corresponding author. The data are not publicly available due to privacy or ethical restrictions.
