# Supplementary material for: Risk Stratification and Adjuvant Chemotherapy for High‐Risk Stage IA Lung Adenocarcinoma: The Unmet Needs
Source: Thorac Cancer. 2024 Dec 21;16(2):e15521. doi: 10.1111/1759-7714.15521 (PMC11735727; doi:10.1111/1759-7714.15521)
Supplement: Supplementary file 1 — Data S1. [file TCA-16-e15521-s001.docx]

Supplementary Table 1. Comparison of Clinicopathological Characteristics Between Patients Receiving ACT and Those Under Observation in the High-risk Group

| Variables | Observation group  (n=416) | ACT group  (n=67) | *p* value |
| --- | --- | --- | --- |
| Male Sex | 219 (52.6) | 39 (58.2) | 0.474 |
| Age, mean (SD) | 59.9 (8.9) | 59.0 (8.6) | 0.199 |
| Ever smoker | 142 (34.1) | 22 (32.8) | 0.835 |
| T1c stage | 267 (64.2) | 45 (67.2) | 0.737 |
| Sol/Mip | 188 (45.2) | 32 (47.8) | 0.795 |
| LVI | 18 (4.3) | 5 (7.5) | 0.347 |
| EGFR positive^†^ | 119 (56.9^†^) | 24 (54.5^†^) | 0.771^†^ |
| ALK positive^†^ | 18 (9.7^†^) | 2 (7.7^†^) | 1.000^†^ |

Data are presented as number (%) unless indicated otherwise.

^†^Calculated among patients receiving testing

ACT, adjuvant chemotherapy; Sol/Mip, solid or micropapillary predominant.

| Author-year | Database | Histological high-risk factors | No. of patients | | Did ACT reduce  recurrence | 5-year survival rate (%) | | Methods for bias control | Reference |
| --- | --- | --- | --- | --- | --- | --- | --- | --- | --- |
|  |  |  | Total | ACT |  | ACT | Observation |  |  |
| Li-2024 | / | Micropapillary presence | 153 | 47 | Yes | 87 | 66 | Not used | 17 |
| Wang-2020 | / | Micropapillary predominant | 152 | 73 | Yes | 84 | 65 | MVA | 18 |
| Liu-2014 | / | Poor differentiation | 20 | NA | Yes | NA | NA | Not used | 19 |
| Luan-2024 | SEER | Poor differentiation | NA | NA | No | 68 | 75 | PSM & MVA | 20 |
| Pathak-2020 | NCDB | Poor differentiation | 10402 | 465 | No | NA | NA | IPTW & MVA | 21 |
| Our study | / | Solid or micropapillary presence | 1328 | 124 | No | 69 | 75 | Risk stratification | / |

Supplementary Table 2. A Literature Review on the Influence of Adjuvant Chemotherapy on Stage IA Non-Small Cell Lung Cancer Patients with Histological High-risk Factors

ACT, adjuvant chemotherapy; MVA, multivariate analysis; NA, not available; SEER, the Surveillance, Epidemiology, and End Results database; PSM, propensity-score matching; NCDB, the National Cancer Database; IPTW, inverse probability of treatment weighting.

**Figure Legends**

Supplementary Figure 1. Study cohort flowchart. LUAD, lung adenocarcinoma; ACT, adjuvant chemotherapy.

Supplementary Figure 2. The calibration curves for predicting 5-year RFS. Nomogram-predicted RFS is plotted on the *x* axis; actual RFS is plotted on the *y* axis. A closer alignment of the drawn line with the diagonal indicates a better calibration model. RFS, recurrence-free survival.
