# Supplementary material for: Risk Stratification and Adjuvant Chemotherapy for High‐Risk Stage IA Lung Adenocarcinoma: The Unmet Needs
Source: Thorac Cancer. 2024 Dec 21;16(2):e15521. doi: 10.1111/1759-7714.15521 (PMC11735727; doi:10.1111/1759-7714.15521)
Supplement: Supplementary file 2 — Figure S1. Study cohort flowchart. LUAD, lung adenocarcinoma; ACT, adjuvant chemotherapy. [file TCA-16-e15521-s003.pdf]

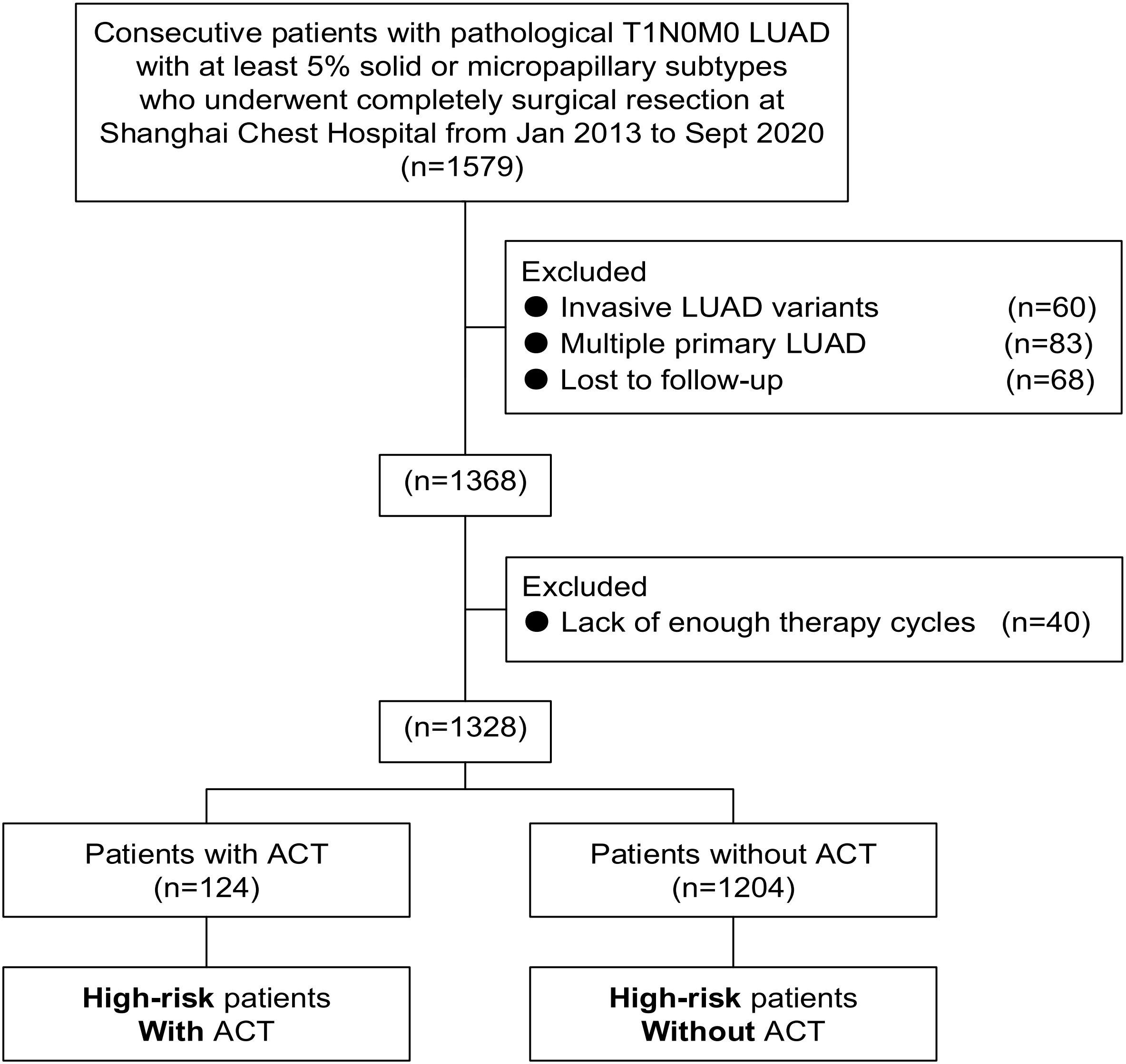
Consecutive patients with pathological T1N0M0 LUAD with at least 5% solid or micropapillary subtypes who underwent completely surgical resection at Shanghai Chest Hospital from Jan 2013 to Sept 2020 (n=1579)
Excluded
Invasive LUAD variants (n=60)
Multiple primary LUAD (n=83)
Lost to follow-up (n=68)
(n=1368)
Excluded
Lack of enough therapy cycles (n=40)
(n=1328)
Patients with ACT (n=124)
Patients without ACT (n=1204)
High-risk patients With ACT
High-risk patients Without ACT
