# Supplementary figures and images for: Risk Stratification and Adjuvant Chemotherapy for High‐Risk Stage IA Lung Adenocarcinoma: The Unmet Needs
Source: Thorac Cancer. 2024 Dec 21;16(2):e15521. doi: 10.1111/1759-7714.15521 (PMC11735727; doi:10.1111/1759-7714.15521)

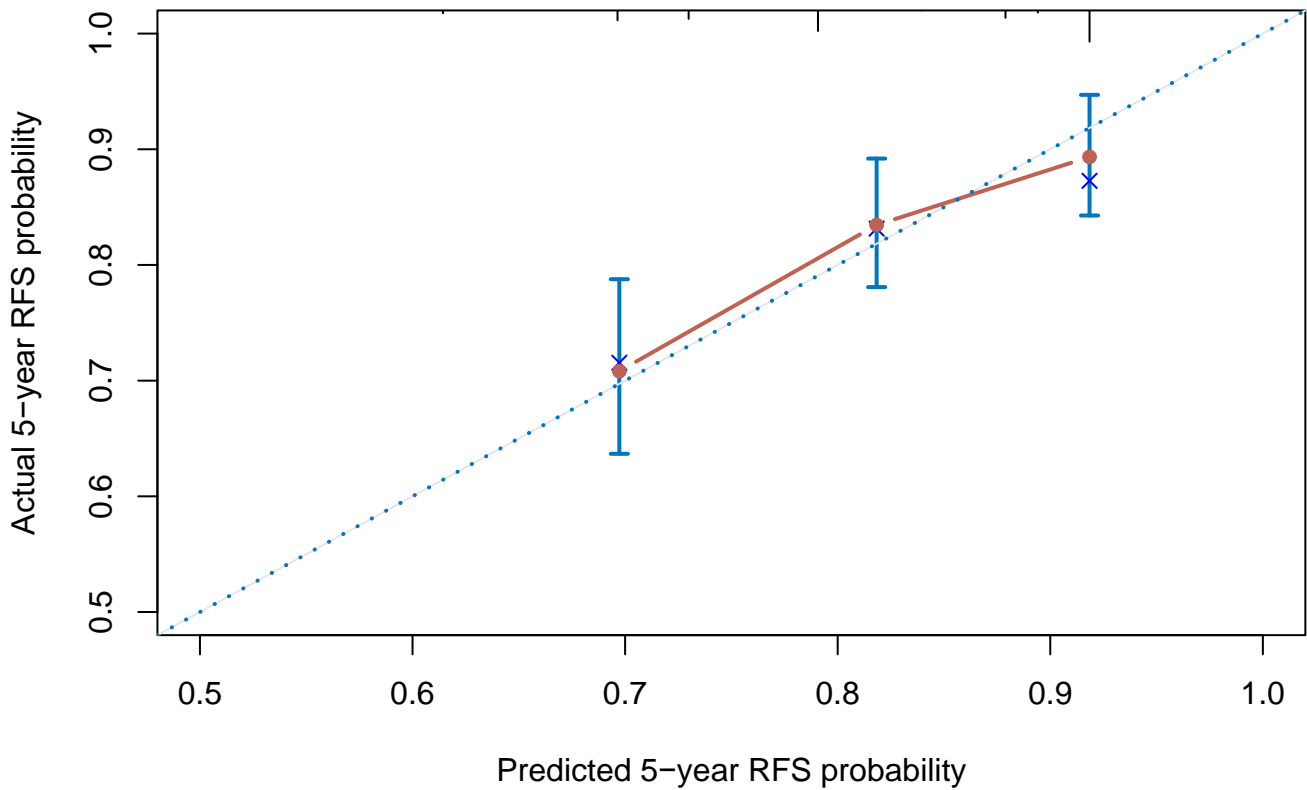

Supplement: Supplementary file 3 — Figure S2. The calibration curves for predicting 5‐year RFS. Nomogram‐predicted RFS is plotted on the x axis; actual RFS is plotted on the y axis. A closer alignment of the drawn line with the diagonal indicates a better calibration model. RFS, recurrence‐free survival. [file TCA-16-e15521-s002.pdf]
